# Sex Differences and Regulatory Actions of Estrogen in Cardiovascular System

**DOI:** 10.3389/fphys.2021.738218

**Published:** 2021-09-28

**Authors:** Kazutaka Ueda, Nobuaki Fukuma, Yusuke Adachi, Genri Numata, Hiroyuki Tokiwa, Masayuki Toyoda, Akira Otani, Masaki Hashimoto, Pang-Yen Liu, Eiki Takimoto

**Affiliations:** ^1^Department of Cardiovascular Medicine, Graduate School of Medicine, The University of Tokyo, Bunkyô, Japan; ^2^Division of Cardiology, Department of Medicine, Johns Hopkins University School of Medicine, Baltimore, MD, United States

**Keywords:** cardiovascular disease, estrogen, sex hormones, cardiovascular homeostasis, non-nuclear signaling

## Abstract

Great progress has been made in the understanding of the pathophysiology of cardiovascular diseases (CVDs), and this has improved the prevention and prognosis of CVDs. However, while sex differences in CVDs have been well documented and studied for decades, their full extent remains unclear. Results of the latest clinical studies provide strong evidence of sex differences in the efficacy of drug treatment for heart failure, thereby possibly providing new mechanistic insights into sex differences in CVDs. In this review, we discuss the significance of sex differences, as rediscovered by recent studies, in the pathogenesis of CVDs. First, we provide an overview of the results of clinical trials to date regarding sex differences and hormone replacement therapy. Then, we discuss the role of sex differences in the maintenance and disruption of cardiovascular tissue homeostasis.

## Introduction

Despite recent advances in medical and interventional therapies, cardiovascular disease (CVD) remains a leading global cause of death in men and women. Although sex differences are well recognized in the epidemiology and outcomes of CVD, their full extent is yet unclear. The results of recently published clinical studies on sex differences may provide new insights into the underlining mechanisms. A recent study that investigated the effect of sacubitril–valsartan on the incidences of cardiovascular death and hospitalization by heart failure (HF) in patients with HF with preserved ejection fraction (HFpEF) reported significantly reduced outcomes in women with HFpEF, but no statistically significant effect was observed in men with HFpEF (Solomon et al., 2019; McMurray et al., 2020). Sacubitril upregulates natriuretic peptide signaling of which cyclic guanosine monophosphate (cGMP) is considered the downstream target (Emdin et al., 2020). Intriguingly, sildenafil, another activator of cGMP signaling, via inhibition of phosphodiesterase type 5 (PDE5), showed sex differences in its beneficial effect on HF in animal models (Takimoto et al., 2005; Sasaki et al., 2014). Studies also showed that women with premature menopause more frequently embrace clonal hematopoiesis of intermediate potential (CHIP), the age-related expansion of hematopoietic stem cells with leukemogenic mutations without detectable malignancy, which is associated with the development of CVD (Jaiswal et al., 2017; Honigberg et al., 2021). Taken together, these clinical and experimental findings suggest clear sex differences in cardiovascular morbidity, natural course and drug efficacy.

The role of sex hormones in the development of CVD, particularly the effect of estrogen on the cardiovascular system, is strongly suggested as the cause of these sex differences. Indeed, several clinical trials, including recent large-scale clinical trials and many basic experiments, have shown the cardiovascular protective effects of estrogen (Bernelot Moens et al., 2012; Schierbeck et al., 2012; Hodis et al., 2016). However, some previous large-scale clinical trials have reported adverse effects of estrogen (Manson et al., 2003; Turgeon et al., 2004), so it seems estrogen may not be entirely beneficial. For clarity in this area, it is necessary to determine the mechanisms of action of estrogen in greater detail. Therefore, in this paper, we first outline the results of clinical trials to date that evaluated the preventive effects of estrogen against CVD, and then, we focus on the molecular function of estrogen signaling in terms of receptors, cell types, organs and pathological models. Finally, we discuss the mechanisms by which estrogen signaling elicits sex differences in the cardiovascular system.

## Sex Differences and Estrogen Hormone Therapy in Cardiovascular Diseases

### Sex Differences in Cardiovascular Diseases

Studies over the decades have reported a distinct pattern of CVD prevalence based on sex. Further, the latest epidemiological report stated that younger women have a lower risk of developing CVD, that the difference between sexes disappears at ages 60–79, and that women overtake men at the age of 80 (Virani et al., 2020), i.e., young premenopausal women have protection against CVDs, and the protection fades away after menopause. Therefore, the cardioprotective role of the female hormone estrogen has been regarded as a major factor responsible for the sex difference in the incidence of CVDs (Vitale et al., 2009).

The overall lifetime risk of HF is similar between the sexes, but sex differences in the epidemiology of HF become apparent when the type of HF is considered. HF with reduced left ventricular ejection fraction (HFrEF) is more common in men than in women (Lee et al., 2009; Dunlay et al., 2017). This type of HF is caused by previous myocardial infarction or dilated cardiomyopathy, and these two diseases are more prevalent in men than in women. In contrast, as revealed by the Framingham heart study, HFpEF is two times more common in women than in men (Lee et al., 2009; Dunlay et al., 2017). Given the fundamental differences in pathophysiology, HFpEF and HFrEF are managed differently. Although results of clinical trials on HFrEF demonstrate the effectiveness of beta blockers, angiotensin converting enzyme inhibitors, angiotensin receptor blockers (ARBs) and sodium–glucose cotransporter-2 inhibitors, these therapies do not definitively decrease morbidity and mortality in patients with HFpEF (Borlaug, 2020). However, there are weak signals of benefit for mineralocorticoid receptor antagonists (Borlaug, 2020). It is important to note that the Prospective Comparison of ARNI with ARB Global Outcomes in Heart Failure With Preserved Ejection Fraction (PARAGON-HF) trial, which is the latest and largest HFpEF outcomes trial, reported a strong sex difference in the efficacy of angiotensin receptor neprilysin inhibitor (ARNI) treatment, with greater benefits observed in women than in men (Solomon et al., 2019; McMurray et al., 2020). Sacubitril–valsartan, compared with valsartan, reduced the prevalence of cardiovascular death and total hospitalizations for HF by 27% in women with HFpEF, but with no effect in men (Solomon et al., 2019; McMurray et al., 2020).

The incidence of ischemic heart disease (IHD) is higher in men than in women throughout their lifespans, even though the sex difference decreases as age increases (Albrektsen et al., 2017). Despite the low prevalence of myocardial infarction in women compared to men, a recent large-scale cohort study showed that women have a higher risk of death and HF than men in the 5 years following an ST-segment-elevation myocardial infarction, even after accounting for differences in angiographic findings, revascularization, and other confounders (Ezekowitz et al., 2020). Women with IHD characteristically have higher prevalence of angina, burden of CVD risk factors, and prevalence of non-obstructive coronary artery disease on angiography than men with IHD (Garcia et al., 2016). Non-obstructive coronary artery disease, also known as microvascular angina, is a disease that predominantly affects postmenopausal women (Jespersen et al., 2012), where estrogen is reported to mediate coronary microvascular function by modulating nitric oxide (NO) in coronary endothelium (Lu et al., 2016; Vanhoutte et al., 2016). CHIP is associated with elevated levels of inflammatory cytokines and accelerated atherosclerosis in animal and human studies (Fuster et al., 2017; Jaiswal et al., 2017; Jaiswal and Libby, 2020). A recent study reported that premature menopause (i.e., menopause before the age of 40), and especially natural premature menopause, is independently associated with increased risk of CHIP (Honigberg et al., 2021). This suggests that CHIP is associated with incident coronary artery disease events in postmenopausal middle-aged women independent of conventional coronary artery disease risk factors.

Although the risk of atrial fibrillation (AF) is higher in men than in women (Ball et al., 2018), it is well documented that women with AF have higher risks of stroke, myocardial infarction and HF than men with AF (Regitz-Zagrosek et al., 2016). In the CHA_2_DS_2_-VASc scoring system used to evaluate the risk of stroke, a point is added for female sex, and patients with total points ≥ 2 who have another risk factor are recommended to receive oral anticoagulant therapy to prevent stroke (January et al., 2014; Kirchhof et al., 2016). Uncontrolled systolic hypertension is a stronger risk factor of incident AF in women than in men, associated with a twofold increased risk of incident AF in women and a 30–60% increased risk in men (Sharashova et al., 2020).

### Hormone Therapy in Cardiovascular Diseases

These sex differences in CVD prevalence may be attributed to estrogen function in cardiovascular organs, and this is supported by studies conducted over previous decades. In 1978, the Framingham study reported that women with surgical menopause have a 2.7-fold higher risk of CVD events than women of the same age without surgical menopause (Gordon et al., 1978). This finding led to the notion that exogenous estrogen could reduce the risk of CVD events in postmenopausal women. Several cohort studies consistently reported the cardioprotective effect of hormone therapy (HT) that lowers risk of CVD (Grodstein et al., 1997; Varas-Lorenzo et al., 2000; Taylor et al., 2020). In turn, major randomized controlled trials reported around the year 2000 showed neutral effects of HT (Hulley et al., 1998; Grady et al., 2002), and a randomized placebo-controlled studies conducted by the Women’s Health Initiative (WHI) reported no benefits in CVD prevention but observed rather increased risks of stroke and deep vein thrombosis (Rossouw et al., 2002). These conflicting results may reflect differences in the time between menopause and the start of HT. Earlier cohort studies have included younger women who underwent HT in the early postmenopausal period, while the randomized studies included participants who received HT 10 years after menopause when responsiveness to estrogen in cardiovascular tissues may have diminished.

In fact, recent studies provided evidence supporting this ‘timing hypothesis’. The WHI-Coronary Artery Calcium Study (CACS) analyzed the calcified plaque burden on coronary arteries in women close to the age of menopause (50–59 years) who received estrogen or placebo. The women who received estrogen were found to have a lower calcified plaque burden than the women who received placebo (Manson et al., 2007). The Danish Osteoporosis Prevention Study (DOPS) was conducted to estimate the effects of early initiated HT on CVD prevention (Schierbeck et al., 2012). In DOPS, healthy women (*n* = 1,006) with a mean age of 49.7 years were randomly divided into two groups: HT group (*n* = 502) and no-treatment group (*n* = 504). Women treated with HT for 10 years had a significantly reduced risk of HF, myocardial infarction and mortality, but they did not have a significant increase in the risk of venous thromboembolism, stroke or cancer (Schierbeck et al., 2012). In the Early versus Late Intervention Trial with Estradiol study (ELITE), participants who had early menopause (<6 years after menopause) and those who had late menopause (≥ 10 years after menopause) were randomized to receive oral 17β-estradiol or a placebo (Hodis et al., 2016). The carotid intima-media thickness (CIMT) measured by ultrasound was the primary clinical outcome as an estimation of cardiovascular risk. 17β-estradiol-treated early menopausal subjects had slower progression of CIMT than placebo-treated subjects, but there was no estrogen effect in late menopausal participants (Hodis et al., 2016). Taken together, these clinical findings suggest that estrogen HT exhibits cardioprotective effects when initiated at an ideal timepoint after menopause, encouraging the researchers to further investigate the molecular and physiological functions of estrogen and estrogen receptor (ER)-mediated signaling in the cardiovascular system.

The effects of sex hormones other than estrogen on CVD have not necessarily been evaluated sufficiently. Progesterone, in combination with estrogen, is effective in inhibiting endometrial hyperplasia and cancer (Beresford et al., 1997). The risk of CVD was lower when progesterone was used in combination with estrogen than with estrogen alone (Grodstein and Stampfer, 1995), suggesting that progesterone may have cardioprotective effects. However, the effects of progesterone itself on the cardiovascular system have been little studied so far. It has also been reported that low serum testosterone levels are associated with an increase of the incidence of CVD in men (Khera et al., 2021), while exogenous testosterone therapy reportedly increases the risk of cardiovascular disease (Basaria et al., 2010; Vigen et al., 2013), so the cardiovascular actions of androgens need to be further studied as well.

## Molecular Mechanisms of Estrogen Receptor Signaling in Cardiovascular Cells

There are two ERs: ERα and ERβ, both of which exhibit high homology (Mendelsohn and Karas, 1999). Ligand-bound ERs translocate from cytoplasm to nucleus and regulate gene expression as transcription factors (nuclear ER signaling). ERs alternatively function without nuclear translocation via enzymatic signaling pathways (non-nuclear ER signaling) (Mendelsohn and Karas, 2010; Ueda and Karas, 2013). Functional ERs are expressed in various cardiovascular cell types of humans and animals, including vascular endothelial cells (ECs), vascular smooth muscle cells (VSMCs), and cardiomyocytes (Mendelsohn and Karas, 1999). Estrogen is also known to signal via a transmembrane G-protein-coupled receptor known as GPER. The characteristics and signaling targets of each ER are summarized in Table 1. Since GPER has been reviewed extensively in other papers (Haas et al., 2009; Prossnitz and Barton, 2011; Feldman and Limbird, 2017; Luo and Liu, 2020), we will focus on ERα and ERβ in this review.

**TABLE 1:** Characteristics of ERs.

ERs	ERα				ERβ		GPER
Identification	1969				1996		1997
Category			Nuclear steroid hormone superfamily				G protein-coupled receptor superfamily
Location	Cytoplasm, nucleus	Membrane (caveolae)		Cytoplasm, nucleus		Membrane (caveolae)	Membrane
Targets	ERE, non-ERE	PI3K, ERK		ERE, non-ERE		ND	AC/PKA, EGFR (PI3K, ERK)
References	(Caulin-Glaser et al., 1997; Lantin-Hermoso et al., 1997; Mendelsohn and Karas, 1999, 2005, 2010; Chambliss et al., 2000, 2010; Simoncini et al., 2000; McKenna and O’Malley, 2002; Florian et al., 2004; Lu et al., 2004; Levin, 2005; Osborne and Schiff, 2005; Pedram et al., 2006; Ueda and Karas, 2013; Ueda et al., 2018)			(Mendelsohn and Karas, 1999, 2005; Chambliss et al., 2002; McKenna and O’Malley, 2002; Patten et al., 2004; Fliegner et al., 2010)			(Haas et al., 2009; Prossnitz and Barton, 2011; Feldman and Limbird, 2017; Luo and Liu, 2020)

*ER, estrogen receptor; GPER, G protein estrogen receptor; ERE, estrogen response element; AC, adenylate cyclase; PKA, protein kinase A; EGFR, epidermal growth factor receptor; PI3K, phosphoinositide 3-kinase; ERK, extracellular signal-regulated kinase; ND, not determined.*

In the nucleus, ligand-bound ERs function as transcription factors, interacting with estrogen response elements, and thereby regulate gene expression (Mendelsohn and Karas, 2005). Also, nuclear ER-estrogen complexes modulate the function of other transcription factor classes via protein–protein interactions. Hence, these complexes control gene expression without directly binding to DNA (Mendelsohn and Karas, 1999; McKenna and O’Malley, 2002). Recruitment of co-activators and displacement of co-repressors differ in each cell type, which determine cellular response to estrogen.

Cellular physiological responses to estrogen are elicited within minutes by the activation of membrane-associated ER, which has been termed “rapid” or “non-nuclear” ER signaling (Ueda and Karas, 2013). Non-nuclear ER signaling has been identified in various cell types *in vitro*, including VSMCs, ECs, and cardiomyocytes (Osborne and Schiff, 2005; Ueda and Karas, 2013). The ERs located in small invaginations of the cell membrane known as caveolae signal the rapid actions via activating kinases or phosphatases to affect cell physiology (Levin, 2005; Pedram et al., 2006). Non-nuclear ER signaling in the cardiovascular system has been most studied in ECs, where rapid (within 15–30 min) activation of endothelial nitric oxide synthase (eNOS) by estrogen was observed (Caulin-Glaser et al., 1997; Lantin-Hermoso et al., 1997). ERs that reside in caveolae activate PI3K, Akt and ERK1/2 kinases, leading to activation of eNOS phosphorylation in ECs (Simoncini et al., 2000; Florian et al., 2004; Pedram et al., 2006). ERα binds to striatin, which is a scaffold protein colocalized with caveolin-1. The activation of PI3K requires that striatin acts as the scaffold protein of the ERα complex at the caveolae (Chambliss et al., 2000; Lu et al., 2004). Blocking ERα-striatin binding, either with a peptide that represents ERα amino acids 176–253 or with the ERα triple-point mutation (lysine 231, arginine 233 and arginine 234 to alanine: KRR), abolishes non-nuclear signaling without affecting nuclear signaling (Lu et al., 2004, 2016; Bernelot Moens et al., 2012; Ueda et al., 2018). Meanwhile, endogenous ERβ was also found in the EC membrane, specifically at the caveolae; however, its associated proteins have not been determined (Chambliss et al., 2002).

## Estrogen Actions in Animal Models of Cardiovascular Diseases

### Ischemic Heart Diseases

In animal models of IHDs, such as myocardial infarction and ischemia–reperfusion, both of ERα and ERβ were reported to play a role in the cardioprotective effects of estrogen. After myocardial infarction, increased mortality and HF exacerbation were observed in global ERβ KO mice (Pelzer et al., 2005). Consistently, cell-type specific overexpression of ERβ in cardiomyocytes improved cardiac function and survival after myocardial infarction. In female mice overexpressing ERα, cardiac fibrosis after myocardial infarction was inhibited with increased angiogenesis (Mahmoodzadeh et al., 2014; Schuster et al., 2016). In an ischemia–reperfusion model, estrogen normalized coronary endothelial dysfunction in ovariectomized wild-type mice, while estrogen failed to reverse it in global ERα KO mice (Favre et al., 2010). ERα KO mice also demonstrated markedly impaired cardiac contractility, increased cardiomyocyte death and mitochondrial damage after ischemia–reperfusion (Zhai et al., 2000; Wang et al., 2006). In contrast, in an *ex vivo* model of global ischemia–reperfusion, the hearts of female ERβ KO mice showed poor functional recovery compared to those of wild-type mice, but no significant difference was observed between ERα KO and wild-type mice (Gabel et al., 2005). Mechanistically, estrogen attenuates reperfusion injuries after ischemia mainly via activation of PI3K-Akt, increased expression of the anti-apoptotic protein BCL-2 and reduced expression of proapoptotic caspase proteins (Patten et al., 2004). In female ERβ KO mice, estrogen treatment failed to induce recovery from ischemic injury or activation of PI3K-Akt signaling in the hearts (Patten et al., 2004; Fliegner et al., 2010). Taken together, ERβ seems to play important roles in cardioprotection against ischemia–reperfusion injury, while the role of ERα varies depending on methodological conditions.

### Cardiac Hypertrophy and Failure

Pathological cardiac hypertrophy develops in response to various pathological stresses, including genetic, mechanical and neurohormonal stress. Excessive and prolonged stress leads hypertrophy to failure. Sex difference is known as a modifier of cardiomyopathy in humans (van Berlo et al., 2013), as well as in genetically modified mouse models of hypertrophic cardiomyopathy, including a missense mutation (R403Q) in the α-myosin heavy chain and a missense mutation (R92Q) in cardiac troponin T (Maass et al., 2004; McKee et al., 2013; Chen et al., 2015). In both transgenic mice, male mice showed an overt phenotype of cardiac hypertrophy and failure compared with female mice (Olsson et al., 2001; Maass et al., 2004; McKee et al., 2013). Importantly, ovariectomized female mutant mice had worse phenotypes with greater impairment of contractile function and myocardial energy metabolism, while estrogen supplementation restored these parameters (Chen et al., 2015). These findings suggest protective effects of estrogen against cardiac hypertrophy and failure.

**FIGURE 1:**
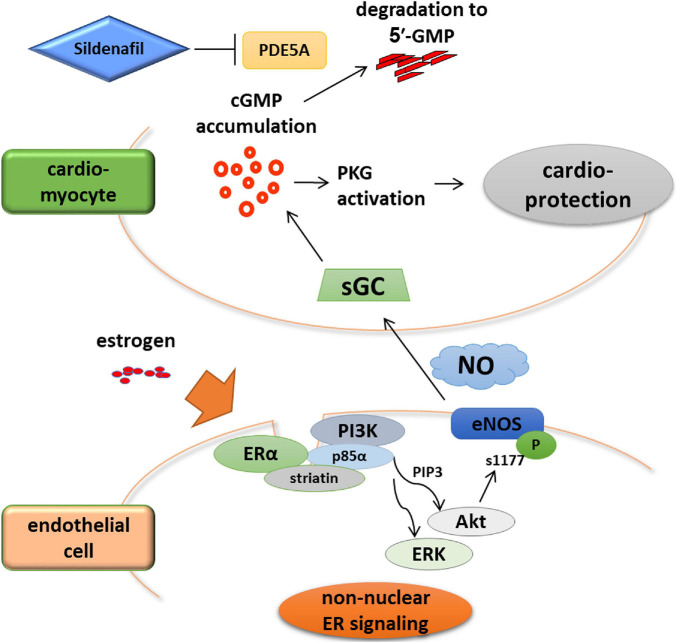
Rapid non-nuclear ERα signaling is indispensable for estrogen to provide NO that activates sGC. ERα non-nuclear signaling requires the interaction between ERα and striatin, a scaffold protein residing at caveolae. A transgenic mouse line in which ERα non-nuclear signaling was selectively disrupted showed that ERα non-nuclear signaling was indispensable to the therapeutic efficacy of cGMP-PDE5 inhibition in heart failure but not to that of sGC stimulation. These data imply the advantage of sGC stimulation over PDE5 inhibition as a potential therapeutic strategy in treating heart failure in post-menopausal women, highlighting the need for female-specific therapeutic strategies.

Results of studies that used global ERα or ERβ KO mice subjected to chronic angiotensin II treatment or pressure overload have suggested the role of ERβ in the protective property of estrogen against cardiac hypertrophy and failure. Mechanistically, the link between estrogen and the cGMP-PKG signaling pathway may be a key that deserves further investigation (Kim and Levin, 2006). Upregulation of cGMP signaling in myocardium has emerged as a novel therapeutic strategy for heart failure, evidenced by recent clinical studies. The Vericiguat Global Study in Subjects with Heart Failure with Reduced Ejection Fraction (VICTORIA) study showed cardiovascular protection by the soluble guanylate cyclase (sGC) stimulator vericiguat (Armstrong et al., 2020). Neprilysin inhibition by ARNI that provides cardiovascular benefits also stimulates cGMP signaling via augmentation of the natriuretic peptides (McMurray et al., 2014). Considering that myocardial cGMP-PKG signaling pathway is deactivated in human HFpEF and that HFpEF is associated with female sex independent of obesity and diabetes (Lee et al., 2009; Dunlay et al., 2017), it is reasonable to assume that estrogen decline and subsequent cGMP deactivation may contribute to the pathophysiology of HFpEF. In fact, estrogen signaling is crucial for a PDE5 inhibitor sildenafil-induced activation of cGMP-PKG in cardiac myocytes to ameliorate HF in female mice (Fisher et al., 2005; Sasaki et al., 2014). Additionally, using a novel knock-in mice, whose ERα are replaced with the ERα harboring triple-point KRR mutation, we recently reported that rapid non-nuclear ERα signaling is indispensable for estrogen to provide NO that activates sGC (Figure 1; Fukuma et al., 2020). These results suggest a potential link between estrogen and cGMP signaling. A recent study provided a great progress in the experimental research of HFpEF, where mice treated with a combination of high-fat diet and inhibition of NOS signaling by L-NAME recapitulates the systemic and cardiovascular features of human HFpEF (Schiattarella et al., 2019). In contrast to observations in humans, however, female mice in the HFpEF model developed a significantly attenuated cardiac phenotype compared with their male counterparts, and this protection in female mice was preserved even by ovariectomy (Tong et al., 2019). Given that ARNI use for HFpEF patients reduced the risk of HF only in women (Solomon et al., 2019; McMurray et al., 2020), extended studies may clarify the molecular mechanisms by which cardiovascular benefits provided by the natriuretic peptide augmentation and its downstream cGMP signaling show the sex difference in HFpEF.

### Injury Response in the Vasculature and Atherosclerosis

Vascular damage provokes regional vascular inflammation and prolonged inflammation leads to pathological vascular remodeling that manifests as neointimal hyperplasia. Estrogen was found to inhibit the intimal thickening in a mouse carotid artery injury model through inhibiting the proliferation of VSMCs and promoting re-endothelialization (Iafrati et al., 1997; Hayashi et al., 2000; Brouchet et al., 2001; Chambliss et al., 2010). In ERα KO mice, estrogen treatment failed to protect vasculature against the vascular injury (Brouchet et al., 2001; Pare et al., 2002), while in ERβ KO mice, it is still protective (Karas et al., 1999; Brouchet et al., 2001), suggesting that ERα is responsible for the estrogen protection on vasculature. The importance of the non-nuclear ER signaling pathway in estrogen-induced vascular protection has been evaluated in gain- and loss-of-function studies. Estrogen dendrimer conjugates (EDC), which was found to specifically bind to membrane ERs but not those in cytoplasm and selectively activates non-nuclear ER signaling, promoted re-endothelialization in injured carotid arteries in an ERα-dependent manner (Chambliss et al., 2010). Notably, endometrial carcinoma cell growth was activated by estrogen, but not EDC, suggesting that selective activation of the non-nuclear ER signaling does not promote cancer growth (Chambliss et al., 2010). In turn, estrogen’s vascular protective effect was not observed in disrupting peptide mice (DPM), in which ERα-striatin binding was disrupted due to overexpression of a peptide that represents ERα amino acids 176–253 (Bernelot Moens et al., 2012), suggesting that non-nuclear signaling plays a substantial role in the protection by estrogen against vascular injury. Meanwhile, ligand-bound ERα mediates the transcription of target genes through the activation function 2 (AF2) domain, which is located on the C-terminal. Knock-in mice without a functional AF2 domain showed impaired estrogen protection against atherosclerosis (Billon-Galés et al., 2011). Conversely, the estrogen effects on re-endothelialization after vascular injury was preserved in these mice (Billon-Galés et al., 2011). Another study using a knock-in mouse model harboring a point mutation of the arginine 264 of ERα (R264A-ERα), in which non-nuclear ERα signaling is selectively abrogated, consistently showed that endothelial healing is mediated by non-nuclear ERα signaling, and in turn, atheroma protection is mediated by nuclear ERα action (Adlanmerini et al., 2020). Additionally, increased atherosclerotic lesion area was displayed in LDL receptor-KO mice transplanted with ERα KO mice bone marrow, suggesting a substantial role of ERα signaling in bone marrow cells for atheroprotection (Ribas et al., 2011).

## Conclusion

Estrogen directly affects cardiovascular tissues and may have considerable influence on the sex differences observed in the epidemiology and outcomes of CVDs. Recent clinical studies have highlighted the diverse cardiovascular effects of estrogen, and research into the mechanisms of action of the sex hormone will be increasingly important in the future.

## Author Contributions

KU and ET wrote the manuscript. NF, YA, GN, HT, MT, AO, MH, and P-YL critically revised the manuscript and contributed to design the figure. All authors contributed to the article and approved the submitted version.

## Conflict of Interest

The authors declare that the research was conducted in the absence of any commercial or financial relationships that could be construed as a potential conflict of interest. The handling editor declared a shared affiliation with one of the authors ET at time of review.

## Publisher’s Note

All claims expressed in this article are solely those of the authors and do not necessarily represent those of their affiliated organizations, or those of the publisher, the editors and the reviewers. Any product that may be evaluated in this article, or claim that may be made by its manufacturer, is not guaranteed or endorsed by the publisher.

## Abbreviations

ARB: Angiotensin receptor blockers
CHIP: Clonal hematopoiesis of intermediate potential
CVD: Cardiovascular diseases
DOPS: Danish Osteoporosis Prevention Study
EC: Endothelial cells
HF: Heart failure
HFpEFHF: With preserved ejection fraction
HFrEFHF: With reduced ejection fraction
HT: Hormone therapy
IHD: Ischemic heart disease
VSMC: Vascular smooth muscle cells
WHI: Women’s Health Initiative.
